# Let's (Not) Talk About Pain: Mothers' and Fathers' Beliefs Regarding Reminiscing About Past Pain

**DOI:** 10.3389/fpain.2022.890897

**Published:** 2022-04-28

**Authors:** Maria Pavlova, Madison Kennedy, Tatiana Lund, Abbie Jordan, Melanie Noel

**Affiliations:** ^1^Department of Psychology, University of Calgary, Calgary, AB, Canada; ^2^School of Public Health, University of Alberta, Edmonton, AB, Canada; ^3^Department of Psychology, Centre for Pain Research, University of Bath, Bath, United Kingdom; ^4^Alberta Children's Hospital Research Institute, Calgary, AB, Canada; ^5^Hotchkiss Brain Institute, Calgary, AB, Canada; ^6^Mathison Centre for Mental Health Research and Education, Calgary, AB, Canada

**Keywords:** function of reminiscing, past pain, parent-child reminiscing, thematic analysis, mixed methods

## Abstract

Children's memories for past pain set the stage for their future pain experiences. Parent-child reminiscing about pain plays a key role in shaping children's pain memories. Parental beliefs about the functions of reminiscing are associated with parental reminiscing behaviors. To date, no studies have investigated parental beliefs regarding the functions of reminiscing about past pain or the associations between parental beliefs and reminiscing about past pain. This study aimed to fill these gaps. One-hundred and seven parents (52% fathers) of young children were asked about their beliefs regarding reminiscing about past pain. Interview data were first analyzed using inductive reflexive thematic analysis. A coding scheme was created based on the generated themes to quantitatively characterize parental beliefs. Parents also reminisced with their children about unique past events involving pain. Parent-child reminiscing narratives were coded to capture parent reminiscing behaviors. Inductive reflexive thematic analysis generated three major themes representing parental beliefs regarding reminiscing about past pain: “reminiscing to process past pain,” “reminiscing as a learning tool,” and “avoiding reminiscing about past pain.” Parents who endorsed avoiding reminiscing used fewer optimal reminiscing elements (i.e., open-ended questions) when reminiscing about past painful experiences with children. Parents who endorsed reminiscing to process past pain used more emotion-laden language when reminiscing about past pain. Mothers and fathers of boys and girls endorsed the reminiscing functions to a similar degree. Parents of older, vs. younger, children endorsed reminiscing to process past pain to a greater degree. Developmental considerations and clinical implications of the findings are discussed.

## Introduction

Pediatric pain is common, occurs across variety of settings [e.g., everyday pains (minor injuries resulting in bumps and bruises), vaccine injections, medical procedures, surgeries], and, despite advancements in pain assessment and management, remains poorly managed (1). To date, research on how language influences child pain has largely focused on the children's immediate painful experience. For example, during painful procedures, reassuring language (e.g., saying to the child “It's okay,” “All done”) has been found to increase children's pain and distress, whereas distracting language (e.g., talking about things other than the pain) reduces it (2).

Children's memories for past pain sets the stage for their future pain experiences. Children who develop negatively-biased memories for pain (i.e., recalling more pain compared to an earlier/initial pain report) following an acutely painful experience (e.g., lumbar puncture, cold pressor task) experienced more pain and distress at the same painful experience in the future (3, 4). Children's memories about past events, whether painful or not, are susceptible to suggestions and reframing (5). However, to date, there have only been three studies harnessing malleability of children's autobiographical memories in the context of needle procedures [for a review see (6)]. These memory-reframing interventions have been delivered by researchers, which has overlooked the key agents of change, children's daily conversation partners, and valuable historians of children's past pain—their parents.

A substantial body of developmental psychology research has demonstrated that parent-child reminiscing, or talking about past events, changes how children remember those events (7). Parental reminiscing about past *pain* differs from reminiscing about other distressing experiences (8), influences how children recall their pain (9), and is associated with children's responses to pain in another person (10). Compared to talking about past sad events, parents reminisced about a past painful event (i.e., their children's recent tonsillectomy) using fewer emotion-related words, fewer explanations, and more coping- and pain-related words (8). In the same sample of young children, parents who used more pain-related words and fewer positive-emotion words had children who recalled more pain, compared to children's earlier pain reports, 1 month after the tonsillectomy (11). In a sample of young typically developing children, parents who used more neutral emotion words (e.g., How did you feel?) when reminiscing about a past painful experience (e.g., everyday or needle-related pain) had children who exhibited more concern about another's pain (10). Together, these findings demonstrate the pivotal role that parent-child reminiscing (i.e., the use of emotion-laden language) about pain plays in shaping children's pain memories and behaviors.

As children develop language, autobiographical memory, and communication skills, they become more competent and actively involved conversation partners. Yet, it is parents who initiate and guide reminiscing with young children. Reminiscing involves *sharing* past autobiographical memories with others; its functions mostly overlap with the functions of autobiographical memory. Specifically, three broad reminiscing functions have been previously identified: social (e.g., foster current or build new relationships, promote a sense of intimacy), directive (e.g., learn a lesson, solve problems, regulate and explain emotions), and defining or maintaining the continuity of the self-concept (12, 13). Kulkofsky argued that the same or similar reminiscing functions would be applicable to parent-child reminiscing (14). Indeed, based on an open-ended measure aimed to elicit maternal beliefs about the functions of reminiscing, Kulkofsky and colleagues identified two key reminiscing functions that mapped onto general reminiscing purposes: (1) reminiscing as a conversational tool to build and maintain relationships (i.e., social function); (2) reminiscing as problem-solving (e.g., regulating emotions, directing children's behavior). The self-concept function was observed less frequently compared to parents using reminiscing to teach children how to remember past events (i.e., memory skills).

Parental beliefs about the functions of reminiscing have been shown to influence their reminiscing behaviors (i.e., content and style of parent-child narrative exchanges) (14). Parents who endorsed the social function of reminiscing [i.e., reminiscing as a way to maintain conversation or having “something to talk about” (14 p. 109)] used more elaborations when reminiscing (i.e., they more frequently asked open-ended questions and provided new information about the past event) and more evaluative statements (i.e., confirmation or negation of child-provided information). Parents who endorsed the directive function of reminiscing (i.e., problem-solving or teaching a child a certain lesson) used elaborations more frequently. These reminiscing behaviors (i.e., the use of elaborations and positive evaluations) have been shown to contribute to optimal developmental outcomes (15). Parental beliefs regarding the functions of reminiscing also were associated with children's emotional issues (16). Specifically, parents who endorsed reminiscing to promote emotional understanding had children with higher levels of emotion problems (i.e., internalizing symptoms) (16).

Little is known about associations between parent sociodemographic variables (e.g., age, gender, education, socioeconomic status) and their beliefs regarding reminiscing. Higher levels of maternal education were associated with lower endorsement of behavioral control, entertainment, and memory skills functions of reminiscing (16). Cross-cultural differences have been reported. Specifically, as compared to Chinese mothers, European American mothers endorsed more reminiscing functions (14).

To date, no studies have investigated the beliefs that parents hold regarding reminiscing about past pain. Given the role of parent-child reminiscing in the development children's pain memories (9, 11) and empathy (10), as well as the associations between parental beliefs about general reminiscing and their reminiscing behaviors (14), it is critical to understand what beliefs parents hold regarding reminiscing about past pain. Indeed, these beliefs would likely influence their actual reminiscing behavior and amenability to memory-reframing interventions. No studies have examined parent sociodemographic characteristics in the context of parental beliefs regarding reminiscing about past pain. This study aimed to fill these gaps. Using a sample of 4-year-old children and one of their parents, we examined the following questions: (1) What beliefs do parents hold regarding reminiscing about past pain and its functions? (2) Are parental beliefs regarding reminiscing about past pain related to parent reminiscing behaviors? (3) Are parent socio-demographic factors (i.e., parent role, age, education) associated with their beliefs about pain reminiscing? Given the dearth of research examining reminiscing beliefs in the context of past pain, no hypotheses were made for the third research question.

## Materials and Methods

### Participants

In total, 395 parents from a database of families, who had previously provided their consent to be contacted for research studies, were contacted via email with the information about the study. Sixty-two present (*n* = 245) of the contacted families did not respond to the recruitment email, and 14 families relocated. One hundred and thirty-six families were screened for eligibility; of those, 20 children were not eligible to participate due to having been diagnosed with a speech delay/developmental disorder. A total of 116 families were enrolled in the larger study described below. Of the 116 participating families, 107 parents completed both tasks (i.e., reminisced about a past event involving physical pain with their children and completed an interview regarding their beliefs about the functions of reminiscing); nine families either could not recall a past event involving pain, or the participating parent did not complete the interview due to time constraints. One parent-child dyad was not able to complete the narrative elicitation task due to the child's anxiety at the lab visit; the child exhibited notable levels of anxiety at the beginning of the lab visit, and the narrative elicitation task was not commenced for that parent-child dyad.

Data from 106 (52% fathers, *M*_*age*_ = 37.3 years) of 4-year-old children (55% girls, *M*_*age*_ = 54.4 months) were included in the analyses (sociodemographic characteristics of the sample presented in Table 1). To recruit an approximately equal number of mothers and fathers, research staff always asked whether a father was available to participate; if a father was available and interested in participating, a preference was given to the father. The participants were recruited as a part of a larger research study that investigated the influence of parent-child reminiscing on children's observed empathy for pain. The aims of this study (i.e., the examination of parental beliefs regarding functions of reminiscing about past pain and the association between parental beliefs about reminiscing and parent reminiscing behavior) are different from previously published data (10, 17). The data reported in the present manuscript have not been previously reported. Parent-child dyads were eligible to participate if (1) the child was 4 years old, (2) one parent provided consent to participate, (3) they could both understand and speak English. Exclusion criteria were a parent-reported language delay and/or a developmental disorder (e.g., Autism Spectrum Disorder). The institutional research ethics board approved this study.

**Table 1:** Sociodemographic characteristics of the sample.

**Sociodemographic characteristic**	***N* = 107**
Parent age (years), M (SD)	37.3 (5.06)
Parent gender (female, % and *n)*	48 (51)
Parent race (% and *n*) Aboriginal Black Chinese Filipino Latin American South Asian White Multiracial	1 (1) 2 (2) 3 (3) 2 (2) 5 (5) 1 (1) 81 (88) 5 (5)
Child's age (months), M (SD)	54.4 (3.7)
Child sex (female, % and *n*)	55 (59)
Child race (% and *n*) Black Filipino Latin American South East Asian White Multiracial Other	1 (1) 1 (1) 2 (2) 1 (1) 77 (80) 16 (17) 2 (2)
Parent highest level of education (% and *n*) High school or less Vocational school/some college College degree Graduate school	7 (7) 22 (24) 50 (54) 21 (22)
Work status (% and *n*) Full time Part time Not employed	70 (75) 17 (18) 13 (14)
Household annual income (% and *n*) $30,000–$39,000 $40,000–$49,000 $50,000–$59,000 $60,000–$69,000 More than $70,000	5 (5) 2 (2) 1 (1) 6 (7) 86 (91)
Parent marital status (% and *n*) Married Common law Single Separated/divorced Do not want to answer	86 (91) 6 (7) 4 (4) 3 (3) 1 (1)

### Procedure

Potential participants were contacted by study staff by email or telephone and provided with information about the study. Once consent was obtained, parents and children visited the research laboratory for a 2-h lab visit. During the visit, parents and children completed a narrative elicitation task by reminiscing about a past painful event. The painful event was chosen by parents and children as a unique autobiographical event in which the child experienced pain (e.g., minor injuries, needle-related pain). The event must have been shared by both the parent and child and must have occurred >1 month prior to the lab visit. In line with previous research (18), the researcher left the room to allow parents and children to talk as they typically would, for as long as they wanted. The parent-child narrative elicitation task was audio-recorded for later transcription and coding. Following the reminiscing task, parents reported their own and their child's sociodemographics. Then, parents completed an interview about beliefs they held regarding reminiscing about past pain. Children received a small toy and t-shirt for participating and parking costs were reimbursed. Parent-child dyads completed other tasks during the lab visit (e.g., children completed an observational empathic behaviors task; parents filled out measures assessing children's empathy). The associated data were reported elsewhere (10, 17).

### Measures

#### Sociodemographics

Parents reported their parent role, their child's sex, their own and child's race, their employment status, the highest level of education, household income, and marital status. These variables were chosen based on the previous research findings that demonstrated an association between parental education levels and culture with reminiscing beliefs (14).

#### Beliefs Regarding the Functions of Reminiscing Qualitative Interview

Using semi-structured interview methods, parents were asked by a trained research assistant about their beliefs regarding reminiscing about past painful events. The interview questions were informed by findings from previous observational research (8) in which parents and children reminisced about two past negative events (i.e., involving sadness or pain), and an interview schedule from the broader developmental literature on the functions of reminiscing (14). The five open-ended questions in the interview schedule (see Supplementary Materials) were designed to elicit rich responses from parents about the perceived value and functions (or lack thereof) of reminiscing about past pain (e.g., *How do you and your child talk about past painful experiences in everyday life? Why do you talk about past pain or why not?*). Research assistants were trained to ask elaborative follow-up questions (e.g., “Tell me more about that”) to prompt parents to provide as much information as possible. Interviews ranged from 2.5 to 15.5 min (*M*_*duration*_ = 6.5 min). Interviews were audio-recorded, transcribed verbatim.

#### Parent-Child Reminiscing Coding Scheme

Parent-child narratives about past pain were transcribed verbatim and broken down into utterances (i.e., conversational turns) using Microsoft Excel. The coding was conducted using an adapted coding scheme drawn from the developmental literature (18) and used in previous research on parent-child reminiscing about past painful events (8, 10, 11). Each parent utterance was coded for structure (e.g., open- or close-ended questions containing new or repeating information; statements with new or repeating information). Parents' utterances were also coded for content (e.g., emotion, pain, and coping words; explanations). Emotion words were further coded by their valence into negative (e.g., *crying, sad, scared*), positive (e.g., *happy, excited*), or neutral (i.e., emotion-related words without explicit negative or positive valence; e.g., *how were you feeling; I was okay*) categories. Consistent with previous research (8, 10, 11, 18), proportions of each code type over the total number of codes used were calculated in order to control for the length of narratives. To avoid potential bias related to researcher-family interactions, the narratives were coded by the first author (MP) who had previous experience using the coding scheme and who did not conduct the lab visits. A random selection of twenty percent of the narratives were coded by an independent reliability coder blind to the study's hypotheses. The inter-coder reliability was strong, ≥0.80 (Cohen's kappa; 0.85 structure, 0.80 content) (19). Discrepancies were resolved by discussion among the two coders until a consensus was achieved.

### Analyses

#### Inductive Reflexive Thematic Analysis

An inductive reflexive thematic analytic approach (20, 21) was used to analyze parental beliefs regarding reminiscing about past pain. Inductive reflexive thematic analysis is a theoretically flexible approach which allows to identify and analyze patterns of themes in a data set (21). Transcripts from the interviews were uploaded and qualitatively analyzed with QSR International's NVivo 12 software (2018) by a primary coder (MK). Interview transcripts were continually and actively read, to ensure familiarization with the data. An iterative qualitative approach was adopted to coding, such that potential themes were revised to determine the final major themes. Throughout the inductive reflexive thematic analysis, frequent consultation and agreement was sought within the study team (MN, MK, MP, TL) to discuss thematic development and interpretation. The following Braun & Clarke's criteria (22) for demonstrating the quality of thematic analysis were met: (1) transcripts were checked against the original audio recordings to ensure accuracy; (2) each data item was given equal attention; (3) data extracts were balanced with analytic narrative; (4) the researchers were positioned as active in the research process; (5) no participants were represented with more than one quotation.

#### Beliefs Regarding the Functions of Reminiscing Quantitative Coding Scheme

Once major themes were generated as a result of the inductive reflexive thematic analysis, the second phase of analysis was conduced; it involved quantitatively coding each parental utterance of the beliefs interview for the presence of the function codes which were created based on the major themes: (1) using reminiscing to process past pain; (2) using reminiscing to teach a lesson; (3) avoiding reminiscing about past pain. If no function was identified for a particular utterance (i.e., a conversational turn that did not address a function of reminiscing about pain and instead provided general information), it received a “null” code (see Supplementary Materials for the Beliefs Regarding the Functions of Reminiscing Coding Scheme). Function codes belonging to each function theme were summed. The first author (MP) coded all transcripts as primary coder, while the secondary coder (MK) coded a random subset (20%) of transcripts. Interrater reliability was calculated with Cohen's kappa (0.89) and was strong (19).

#### Quantitative Analyses

Quantitative data were analyzed using the Statistical Package for the Social Sciences, version 27 (23). Pearson correlations between parental reminiscing beliefs and parent use of reminiscing codes were performed. *T*-tests were conducted to examine differences in endorsed parental beliefs regarding reminiscing as a function of sociodemographics (i.e., child's sex, parent role, education, race, income).

## Results

### Qualitative Analysis

Thematic analysis generated three major themes, entitled “reminiscing to process past pain,” “reminiscing as a learning tool,” and “avoiding reminiscing about past pain.” Each theme is supported with quotations. To protect confidentiality, identifying participant name and location information has been removed. The parent role of each participant has been indicated.

#### Theme One: Reminiscing to Process Past Pain

The first theme captures parents using reminiscing about past pain to process past pain to contextualize and normalize painful experiences, and to foster children's coping skills, resilience, and empathy for pain.

Some parents utilized reminiscing as a way to provide some perspective around a painful event for their child. Parents recognized that young children may not understand the severity of their pain and how to manage it. Reminiscing about a past painful event served as a reference point for current or future painful experiences. Parents also reminded their child about a past painful event they had successfully overcome and identified similarities/differences between previous and current experiences as a way of providing a recognizable anchor for the child to link the current/future and past situations.

“*(I consider how) the past painful experience relate(s) to the current one. And the similarities that they share as a way of explain(ing) it to him, that it might get better like it got better last time…so, I'd say ‘Hey, last time this was painful, but now remember that it got better.”' (Ryan, father)*

Due to the ubiquitous nature of pain in early childhood, several parents noted the importance of normalizing painful experiences through reminiscing. Past painful experiences illustrated that pain is an everyday part of life, which, in turn, helped children to accept the normalcy of occasional pain experiences and to learn that pain is not to be feared.

“*But most of the time, if I'm bringing it up, maybe something else painful has happened and we'll say like, ‘Hey, remember that time when (you got hurt) and you were ok.' … You want them to learn resilience, learn that things can be bad but you can be better.” (John, father)*

Parents reminisced with their children to help children better cope with future painful experiences and foster resilience. Joint reminiscing about past pain contributed to developing and maintaining supportive parent-child relationship, which enhanced children's resilience. Similarly, reminiscing helped children to (re)create and (re)frame narratives about their past painful experiences, which added to children's evolving sense of self. Further, by talking about a past painful experience, parents reminded their children about coping strategies that had worked for them before and encouraged their child to rely on them in the present and future. The successful coping strategies contributed to children believing that are able to deal with pain.

“*Trying to come back around to, ‘Yeah sometimes things are bad, but you're strong, resilient, you'll get past it.' Try and instill that whole ‘You got this!' mentality. ‘You will be alright to move past it!'. So, I think in a way of bring(ing) up (past pain), it can be a pro to…demonstrate to them the virtues that you are trying to instill. The resilience. Talking it out instead of feeling distressed about it. Seeking solutions and stuff, settling on issues.” (Peter, father)*

According to parents, reminiscing with a parent encouraged children to express their feelings about a past painful incident, as well as resolve and regulate residual emotions. Parents processed pain-related emotions together with their children, which involved identifying, understanding, and regulating emotions. Further, parents also highlighted the benefit of reminiscing, as it provides a way to process the past painful experience when the stress of the ongoing pain subsides or resumes. The latter implies that parents may see reminiscing as a way to process past pain only in certain circumstances (i.e., when the pain is resolved/subsided).

“*I think one of the reasons we would (reminisce about pain)….is to help her examine the event from a place where she is now in a better state about it, where she is not emotional about it anymore, she is neutral. And I think it's important to reflect on um, on our feelings and understand our feelings when we are capable of managing those thoughts.” (Anna, mother)*

Several parents used reminiscing to encourage their children how to feel sympathy and respond empathically to pain in others (e.g., siblings, parents, friends). Discussing past salient painful events reminded children how they were feeling and what they were experiencing, which, in turn, helped children to better understand the other person's pain experiences. In this instance, reminiscing provided an opportunity for children to learn to recognize and respond appropriately to pain in others.

“*Some reasons [to reminisce] would be to encourage sympathy or empathy for other people. To be able to take her experiences and understand how other people might be feeling. Either just someone who happens to be in pain or not feeling well or in a case where maybe she has hurt someone – her sister. To kind of explain…put in perspective what the other person might be feeling.” (Sam, father)*

Taken together, this theme captures parents' view of reminiscing about past pain as a way to process past pain, make sense of it, and foster resilience and empathy. To achieve this, parents use past events involving pain to provide a reference point for any current pain and/or to highlight the ubiquity and normative/everyday nature of pain. Reminiscing about past pain also allowed parents to process any pain-related emotions and emphasize pain coping skills that children used in the past. Finally, by processing past pain parents had an opportunity to underscore the importance, value, and specific examples of empathic responses to pain.

#### Theme Two: Reminiscing as a Learning Tool

The second theme concerns parental beliefs regarding the learning value of reminiscing about past pain. Parents reflected on their use of past painful experiences as a way to teach their children to prevent or prepare for future pain. Parents sometimes also added warning reminders about past painful experiences to alert and dissuade their children from dangerous activities.

Some parents used reminiscing about past pain as a “learning tool” to prevent a potential future painful experience by “reinforcing a lesson” to be learned from the past. This typically occurred in the context of everyday pains (e.g., minor injuries resulting in bumps, scrapes, or bruises) and injuries (e.g., broken bones). Talking about a past painful event that was similar to the current situation reminded children that specific behaviors may result in an injury. According to parents, reminiscing encouraged children to consider potential risks and behave more cautiously.

“*You can remember what you've experienced and use it to learn for the future. […] You would use your past experiences to learn and to make better choices in the future and so he can avoid getting hurt.” (Karen, mother)*

Parents used reminiscing to prepare their children for predictable future painful events (e.g., vaccine injections, dental or medical procedures). Parents reminded children how it felt when they experienced pain the previous time to reduce anticipatory pain-related fear and distress during future painful procedures. Some parents highlighted the distressful aspect of pain-related fear and wanted to reduce the fear by reminding children that past fear dissipated. Preparation for future painful events was especially applicable for medical procedures, such as vaccine injections, that are commonly associated with fear. In preparation for an upcoming vaccination, parents reminisced about previous vaccine injections to alleviate fears and anxiety by reminding their child that the pain had been temporary.

“*His flu shot—he didn't like it last year and so we were starting to talk about it again for this year. And preparing him – ‘it was only one second, and Daddy got one, and [sibling's name] got one, and it was over before you knew it even though it hurt, right?” (Polly, mother)*

Several parents endorsed using reminiscing about a past painful event to warn their children to avoid engaging in unsafe behavior (e.g., climbing furniture). While teaching children to prevent pain involved discussing *actions* that could lead to a painful outcome, the warnings frequently focused on how the pain *felt* to discourage unsafe behaviors. Some parents reminisced about a past painful experience to prompt a salient memory of a painful sensation and discourage a behavior by tying it to a consequence.

“*If she had gotten hurt physically then I will say… ‘Oh, remember when you fell down here? We don't wanna do that now, so make sure you- you take these precautions.' …I think that's how I would talk about pain just to… avoid the pain.” (Nina, mother)*

Overall, this theme captures parental use of reminiscing about pain to teach a lesson of preventing unnecessary pain or preparing for predictable pain (e.g., a painful medical procedure). To make the lesson salient and persuasive, some parents infused their reminiscing about past pain with explicit warnings and reminders about how past pain *felt* to encourage their children into avoiding dangerous activities.

#### Theme Three: Avoiding Reminiscing About Past Pain

The final theme captures parental avoidance of reminiscing about past painful events. For some parents, reminiscing about past pain lacked value and brought their children unnecessary distraction from the present (pain-free) moment. Reminiscing about past pain also implied direct harm by bringing back the fear and stress of past pain, which was in direct contradiction with parents' duty to protect their children from harm and pain. Contrastingly, other parents did not believe that painful events would have the same impact as other types of events that occur in childhood (e.g., sad or scary events), thus perceiving no value in reminiscing about past painful events.

“*It's…easier to talk about a sad event and just kind of bring it up again...to try to build that resilience for when sadness happens, whereas pain you can't predict when it's gonna happen and it kind of overrides everything. So, it's hard to talk about. I don't see the value as much either.” (Paul, father)*

Some parents also described developmental barriers to reminiscing with their child; they believed their child was too young to benefit from reminiscing about pain and may not understand what the parent was trying to convey. In these instances, parents did not discredit the value of reminiscing generally, but rather indicated that they would prefer to address past painful events when the child was older and better able to articulate themselves.

“*I don't think it's gonna benefit her to talk about the pain at this age. I think when she gets older, for sure, I'll be sitting down and talking about the pain and…especially like if she ever had a broken arm. […] I don't want to talk about it to her, but I know when she does get older, I will be talking about pain and how to deal with pain.” (Sarah, mother)*

Several parents expressed that once the painful experience was over, there was nothing further to address with their children. They would discuss pain “in the moment” or “as needed” but found it necessary to “move on.” “*I guess once it's dealt with, it's just sort of done. And we don't tend to…speak about it again.” (Alicia, mother)*. In such instances, parents believed that when pain is out of sight, it should be (and is) out of mind.

Some parents believed that discussing a past painful event would scare their child and/or have long-term effects. Some parents described that bringing up a past painful event would cause their child to relive the past painful experience, which is unnecessary and may be harmful.

“*The painful things I think, is sometimes tough to talk about with her…If it's fairly minor it brings it back up and then it's like it's all over again…Like if she falls and scrapes her knee or something, then it's really in the grand scheme of things fairly minor and you know she probably doesn't feel it anymore. But if we bring it up again, it's like it's happened all over again and…we go through the process of putting band-aids on it again... And so, it's like we know it's not still painful…[but] it makes them relive it almost…even after the pain is not physically present anymore.” (Gregory, father)*

Some parents perceived reminiscing about a past painful event would cause their child to re-experience pain and fear, providing a stark contrast to the perceived role of parent as protector from any negative experiences. Parents often acknowledged a tension: it was not realistic to prevent their child from experiencing *any* pain; however, by avoiding reminiscing about past painful events, parents could reduce the amount of children's exposure to pain and fulfill their protective parental role.

“*Your focus is to protect them from pain, right? So, reliving pain, for whatever reason, doesn't feel like you're protecting them. At the same time, you know, you have to equip them for (the fact that) life isn't fair and that you're gonna have painful experiences.” (Jim, father)*

In sum, this theme captures parental avoidance of reminiscing about past pain. The lack of value of revisiting past pain was brought on by parents' desire and duty to protect children from pain. Underlying this desire is the belief that *talking* about past pain may harm children by triggering the sensations of pain and associated emotions (e.g., fear). By contrast, focusing on the present moment and denying any influence of past pain was perceived as a safer option that is more in line with the parental duties.

### Quantitative Analyses

#### Association of Parental Beliefs About Functions of Reminiscing and Reminiscing Behaviors

On average, parents endorsed beliefs in the processing function of reminiscing 1.9 times (*SD* = 2.0, *range*: 0–11), beliefs in reminiscing as a learning tool 2.1 times (*SD* = 2.0, *range*: 0–9), and beliefs in avoiding reminiscing 1.4 times (*SD* = 1.6, *range*: 0–8). Null codes (i.e., general information that did not contain beliefs about reminiscing functions) were most frequent (*M* = 10.4, *SD* = 1.8, *range*: 3–36). Correlations between parental beliefs about functions of reminiscing and parent use of reminiscing codes, as well as descriptive statistics for reminiscing codes, are summarized in Table 2.

**Table 2:** Correlations between parental beliefs about functions of reminiscing and parent use of reminiscing codes.

	**M (SD)**	**Reminisce to process past pain**	**Reminisce to learn a lesson**	**Avoiding reminiscing**
Reminiscing structure codes				
Open-ended elaboration question	0.04 (0.05)	0.01	0.04	0.08
Open-ended repetition question	0.12 (0.07)	−0.05	0.06	−0.20^*^
Yes-no elaboration question	0.27 (0.10)	0.02	−0.01	0.07
Yes-no repetition question	0.21 (0.12)	−0.17	0.10	0.14
Statement elaboration	0.15 (0.14)	0.09	−0.04	0.01
Statement repetition	0.04 (0.05)	−0.02	−0.01	−0.11
Evaluation statements	0.17 (0.09)	0.10	−0.10	−0.10
Reminiscing content codes				
Positive emotion	0.04 (0.08)	0.03	−0.04	0.12
Negative emotion	0.11 (0.12)	0.21^*^	−0.12	−0.05
Neutral emotion	0.04 (0.07)	0.04	−0.05	−0.01
Pain	0.59 (0.21)	−0.13	0.04	−0.02
Coping	0.12 (0.14)	0.01	<0.01	−0.03
Explanation	0.10 (0.11)	<0.01	0.09	0.04

*N = 106*;

* *p < 0.05 (two-tailed)*.

Parents' belief in the processing function of reminiscing was significantly associated with parental belief in reminiscing as a learning tool, such that the more parents endorsed reminiscing to process past pain, the less parents endorsed using reminiscing about past pain as a learning tool, *r* = −0.41, *p* < 0.001.

Parents who endorsed avoiding reminiscing about past pain used open-ended questions less frequently, *r* = −0.21, *p* = 0.03. There were no other significant associations between parental beliefs about functions of reminiscing and parental use of structural reminiscing codes (e.g., use of closed-ended questions, use of elaborative statements).

Parents' belief in the processing function of reminiscing was associated with parent use of words associated with negative emotions. Specifically, the more parents endorsed reminiscing to process past pain, the more frequently they used negative-emotion laden words (e.g., sad, crying) when reminiscing with their children about past pain, *r* = 0.22, *p* = 0.02. The other associations between parental beliefs about functions of reminiscing and parent reminiscing content were not significant.

#### Individual Characteristics and Parental Beliefs About Functions of Reminiscing

Mothers and fathers did not differ in their endorsement of different functions of reminiscing, *p*s >0.05. Similarly, parents of boys vs. girls did not differ in their endorsement of different functions of reminiscing, *p*s > 0.05.

Parents' age did not have a significant association with parents' endorsement of different functions of reminiscing, *p* > 0.05. However, parents of older children were more likely to endorse the processing function of reminiscing, *r* = 0.21, *p* = 0.03. Parental beliefs about different functions of reminiscing did not differ as a function of their race or education, *p*s > 0.05.

## Discussion

This is the first study to examine parental beliefs regarding the functions of reminiscing about past painful events. Three key themes were identified as a result of the inductive reflexive thematic analysis: “reminiscing to process past pain,” “reminiscing as a learning tool,” and “avoiding reminiscing about past pain.” Based on the thematic analysis, a coding scheme was created to quantitatively characterize parental beliefs regarding the functions of reminiscing about past pain. The quantitative coding allowed us to examine the associations between parental reminiscing beliefs and reminiscing behaviors (i.e., how parents reminisce about past pain with their children). The more parents endorsed avoiding reminiscing about past pain, the less frequently parents used elaborative reminiscing elements (i.e., open-ended questions). The more parents endorsed reminiscing to process past pain, the more frequently they used words associated with negative emotions. Parental beliefs regarding the functions of reminiscing about past pain did not differ as a function of their sociodemographic characteristics. However, the older their children were, the more parents endorsed reminiscing as a way to process past pain.

### Qualitative Results

The identified functions of parent-child reminiscing about past pain overlap with the previously reported functions of general parent-child reminiscing. The first function of reminiscing to process past pain maps onto both social and problem-solving (specifically, emotion regulation) functions of general parent-child reminiscing. Indeed, both memory sharing and pain experiences are inherently social (24). Parents shared that reminiscing about past pain promotes the development of empathy for others' pain, which, in turn, may enhance children's social skills and relationships. These beliefs are in line with existing research. In a sample of young children, elaborative and emotion-rich reminiscing about past pain was associated with children's observed concern for another person's pain (10). Another study demonstrated that recalling one's autobiographical past painful experiences elicited higher levels of empathy toward another person's chronic pain, as compared to recalling a movie character in pain (25).

Within the first theme of reminiscing to process past pain, parents also reflected on reminiscing as a way to contextualize the pain experience by contrasting pain in the moment with a painful incident that occurred in the past. Thus, reminiscing about past pain serves as a reference point for present or future pain experiences and may help children to regulate pain-related emotions. Using reminiscing about past pain to normalize pain in everyday life similarly serves emotion-regulating and social (i.e., pain happens to everyone) functions. Finally, parents emphasized that processing past pain by the means of reminiscing may help children develop pain-specific coping skills and foster resilience. Importantly, reminiscing usually takes place in safe settings once the stressful experience and associated intense emotions are over (15). This kind of temporal and physical separation from the distressing experience creates an optimal environment to understand distressing emotions, learn to regulate them, infuse the memory of the distressing experience with positive emotions, and discuss helpful coping skills to use in future similar situations. Of note, the first theme of reminiscing to process past pain straddles two general reminiscing functions (i.e., social and directive/problem-solving), which may be due to multidimensional nature of pain that encompasses sensations, emotions, behavioral responses, and social interactions.

Parent-endorsed social and emotion-regulating functions of reminiscing about past pain are in line with the previously established benefits of parent-child reminiscing. Children whose parents discuss past events using elaborative elements (e.g., open-ended questions, novel details, emotion-related words) have a better-developed self-concept, understanding of emotions, and social competence [for a review, see (15)]. Further, the use of elaborative elements when reminiscing about *past pain* has been associated with more accurate/less distressing pain memories in young children (9, 11), greater children's concern for another person's pain (10), and better developed general episodic memory (26).

The second function of reminiscing about past pain as a learning tool mirrors the problem-solving (or directive) function of general parent-child reminiscing. The directive function of general reminiscing is future oriented as it serves to prepare children for a planned event or a future action (14). Similarly, parents reflected that reminiscing about past pain may be used to teach children a lesson about how to prevent or prepare for pain in the future. Additionally, talking about past pain may serve as a warning for children not to engage in dangerous activities. The latter belief is directly in line with the evolutionary function of pain. It has been argued that pain evolved to guide adaptive behavior and escape danger (27). Thus, reminiscing with children about past pain as a way to remind them about the unpleasant consequences of dangerous/risky behaviors (i.e., pain) has some evolutionary merit. At the same time, warning children and reminding them about the danger, unpleasant sensations and emotions (e.g., fear) associated with pain could also foster maladaptive avoidance and fears.

Parents' beliefs that reminiscing about past pain may function as prevention of or preparation for future pain align with the existing theoretical models of preparatory interventions in procedural pain [for a review, see (28)]. Preparing children for painful medical procedures often employs, relies on, and/or has to consider children's memories for past pain of a similar nature [for a review, see (29)]. Parent beliefs that reminiscing about past pain may serve as a useful lesson in preventing and/or preparing for future pain may fuel parental willingness to participate in preparatory interventions with reminiscing elements [for a discussion, see (29)].

The third theme, avoiding reminiscing about past pain, is unique to reminiscing about past pain and has not been endorsed in previous studies on parental beliefs regarding the functions of reminiscing more broadly. When reflecting on the reasons for avoiding reminiscing about past pain, some parents shared that they did not see any value in reminiscing about past pain and found it to be developmentally inappropriate to discuss pain with young children. This is a notable belief given that is not supported by existing research. For example, children as young as 3 years show empathy for another person's pain (30), can describe pain and explain its causes (an ability that increases with age) (31), and can remember and report past pain [for a review, see (32)]. Thus, developmentally, young children are capable of talking about their past painful experiences. Further, not engaging in reminiscing about past pain may forego one of the central benefits of reminiscing: making sense of the past experiences and processing associated emotions (e.g., fear). Left unprocessed and undiscussed, these distressing memories and emotions may contribute to persistent pain-related fears [e.g., needle fears that affect up to 60% of children (33)].

Another reason for avoiding reminiscing about past pain relates to parents' preference to focus on the present moment, as opposed to revisiting the past pain that has ended and is perceived by these parents to no longer influence their children. Yet, theoretical models (34) and empirical studies (3) confirm that the experience of pain is not over once the sensory aspect of pain ends. Emotions associated with pain (e.g., fear, anxiety) may linger and contribute to how children remember their past pain (35). Further, children's pain memories are a more robust predictor of future pain experiences compared to the initial reports of pain. Up to 25% of children who remember their pain as being worse than it actually was are at risk of experiencing higher levels of pain in the future (4). Thus, parents' preference to focus on the present moment may need to be addressed and adjusted to be in line with the current state of research.

Some parents also highlighted that reminiscing about past pain may ignite fear in their children. In line with those beliefs, several parents emphasized that reminiscing about past pain would contradict their desire and duty to protect children from pain. No research examined the association between reminiscing about past pain and immediate pain-related emotions. However, when parents reminisced about children's past pain experiences using more frequent use of pain-related words, their children remembered past pain in a negatively biased/distressed way (11). The same study found that discussing positive emotions experienced during the past painful experiences led to the development of more accurate/less distressing memories for pain (11). Parents' desire to protect children from (past) pain may be reframed by highlighting the benefits of reminiscing about past pain and the possibility to focus on the positive aspects of pain experiences. Further, existing research supports the benefits of discussing negative emotions associated with past events; reminiscing about past negative emotions provides opportunities for validation and supports optimal developmental outcomes (15).

### Quantitative Results

Parental beliefs about the functions of reminiscing were associated with some of the parents' reminiscing behaviors (i.e., reminiscing style). Specifically, the more parents endorsed avoiding reminiscing about pain, the fewer open-ended questions (an elaborative reminiscing element) they used. The more parents endorsed reminiscing to process past pain, the more frequently they used negative emotion-laden words when reminiscing about past pain. Previously, Kulkofsky and colleagues (14) demonstrated that parents who used reminiscing a way to engage their children in a conversation (i.e., endorsed the social function of reminiscing) used more elaborative reminiscing elements, which are, in turn, associated with and predict better-developed memory skills in children (36). Similarly, emotion-rich reminiscing about past pain with the abundance of open-ended questions was predictive of accurate/less distressing pain memories in young children undergoing tonsillectomy (11). In sum, parental beliefs about the functions of reminiscing about past pain were, to an extent, associated with how parents engage (or not) in reminiscing with their children.

Of note, however, is the finding that most associations between parental beliefs about function of reminiscing and reminiscing codes were not significant indicating that not all reminiscing beliefs and behaviors are connected. It is possible that parents hold implicit or unconscious beliefs about reminiscing functions that guide reminiscing behaviors. Future studies should assess the implicit beliefs and their role in parent-child reminiscing. It is also important to note that parental endorsement of avoiding reminiscing about past pain may not automatically imply that a parent is fervently against talking about pain and has never engaged in it. The avoidance of reminiscing about pain may exist on a continuum from a neutral and ambivalent attitude toward reminiscing to a strong belief in the potential harms of reminiscing about past pain. Future research may examine the intensity of parental beliefs regarding the functions of reminiscing about past pain, as well as their associations with parents' beliefs about general reminiscing. It is equally important to consider that avoiding reminiscing about past pain may be helpful and/or culturally bound. Functions of parent-child reminiscing differ across cultures. For example, European American mothers endorsed problem-solving functions of reminiscing more frequently than Chinese mothers (14). As argued by Kulkofsky and colleagues, Chinese mothers may consider reminiscing to be too removed from a situation that required problem-solving and opt to teach children a lesson in a more formal context without references to the past (14). Thus, in some cultures, pain prevention and warnings against hazardous behaviors may occur outside of the reminiscing context.

Given the above-described association between parent beliefs about functions of reminiscing and their reminiscing behaviors, as well as the key role of parent-child reminiscing in children's development (15), it is important to examine factors that are associated with parents' beliefs about functions of reminiscing. In the present sample, mothers and fathers, as well as parents of boys and girls, did not differ in their beliefs regarding different functions of reminiscing about past pain. These findings contrast previously demonstrated associations between parent-endorsed reminiscing functions and child's sex. Mothers of daughters, but not sons, reminisced to develop memory skills and peer relationships (16). Yet, in the context of reminiscing about past *pain*, few differences as a function of parent role and/or child's sex have been demonstrated (8, 9, 11, 17). Considering the gendered portrayal of pain in children's popular media (37), as well as sex and gender differences in pediatric chronic pain (38), it is possible that differential beliefs about reminiscing about past pain may become more apparent as children age.

Similarly, parents' race, age, or education were not associated with their beliefs regarding reminiscing about past pain. The lack of significant findings, specifically associated with parents' race, in the present study may be due to the homogeneity of the sample. Previous research demonstrated a marked influence of parents' culture on parental beliefs about the purpose of reminiscing. For example, European American, as compared to Chinese, mothers were more likely to endorse social and problem-solving functions of reminiscing (14). Previous research also demonstrated that mothers with higher levels of education did not endorse memory skills, behavioral control, and entertainment functions of reminiscing as highly as mothers with lower levels of education (16).

Children's age was positively correlated with parental endorsement of reminiscing to process past pain. Parents may deem talking about pain—past, future, or present—as developmentally inappropriate for younger children. As children grow older, develop their cognitive abilities, and face higher socio-emotional demands, pain processing (i.e., a social and emotion-regulating function of reminiscing about past pain) becomes a more appropriate and needed function of reminiscing. Kulkofsky and colleagues reported a similar trend with mothers of older children endorsing reminiscing to promote peer relationships (16). The observed association between children's age and the social purpose of reminiscing may also be a function of previously observed parents' tendency to discuss past emotions and provide explanations to past events (both distressing and positive) with older children (18). Similarly, when reminiscing about a past surgery, parents used emotion-laden language more frequently with older children (8). However, the association between age and parental reminiscing beliefs is qualified by the restricted age range (i.e., the sample included only children aged 4 years). To further examined the association, future studies should include children of different ages.

### Developmental Considerations and Clinical Implications

It is important to consider the present study's findings within a developmental context. Children learn about pain through personal experiences starting from the first days of life (39) and social observations (e.g., observing caregivers' reactions) (40). By the age of 3 years, children can describe their pain and identify its cause (31). As children age, their ability to describe pain and to identify its cause and value becomes better developed (31). Thus, for young children, parents may play a key role in explaining pain's causes and purpose, as well as discussing ways to cope with pain. Using reminiscing to explain children's own past painful experiences and to contextualize pain may be one of the ways to support and organize young children's understanding of pain.

Children's empathy for pain follows a developmentally distinct trajectory as compared to empathy for negative emotions (i.e., sadness) (30). Children aged 18 months and 36 months reacted to another person's sadness similarly; when witnessing another person's pain, younger, vs. older, children were less concerned about it (30). Younger children may benefit from parents scaffolding their empathy for pain. Thus, parental beliefs regarding reminiscing to process past pain as a means of (pain) empathy development may be particularly important earlier in developmental trajectories.

The present findings have clinical implications. Given the association between endorsing the processing function of reminiscing about past pain and optimal reminiscing behavior (i.e., use of emotion-related words), it would be important to assess and, if needed, modify parental beliefs about the functions of reminiscing. Inquiring about parental beliefs regarding discussing past pain and the role of pain memories in children's pain experiences is a part of a parent-led memory-reframing intervention (9). Future iterations of the intervention may focus more on parental beliefs about the functions of past pain reminiscing. If a parent believes that reminiscing about past pain is harmful, that parent may not fully participate in and/or adhere to the intervention principles. Future studies may also examine the malleability of parental beliefs. Parent reminiscing style is amenable to interventions (9, 41); parents' beliefs about reminiscing functions may, therefore, also be malleable. Given the strong associations between parent reminiscing behaviors and children's developmental outcomes (42), parent reminiscing style has been (9, 41) and should continue to be a key target in reminiscing interventions. Future randomized studies may examine whether reminiscing behaviors may change as a result of altering parental reminiscing beliefs.

### Limitations

The study should be viewed in light of limitations that may direct future research. First, the sample was mostly White and of middle and upper-middle socioeconomic backgrounds, which may limit the generalizability of findings. Existing research demonstrated marked differences in both parent-child reminiscing and parent beliefs about the functions of reminiscing as a function of parents' culture (14, 43). Future studies would benefit from including more diverse samples to examine any cultural differences in parent-child reminiscing about past pain, as well as parental beliefs regarding the functions of reminiscing about past pain. Second, a limited number of sociodemographic variables (i.e., parent role, age, education) were examined as potential predictors of parental beliefs regarding reminiscing functions. Parents' and children's attachment status may be contributing to both parent-child reminiscing behaviors and parental beliefs about it. Indeed, elaborative parent-child reminiscing has been linked with secure attachment style (44, 45). It is, therefore, possible that parent attachment style may be influencing parent beliefs about the value (or lack thereof) of reminiscing about past pain, such that securely attached parents may be endorsing reminiscing to process past pain more compared to insecurely attached parents. Third, the quantitative assessment of parental beliefs regarding the functions of reminiscing about past pain was created for the current study and has not been validated against existing questionnaires that assess parental attitudes toward functions of reminiscing (e.g., Caregiver–Child Reminiscence Scale) (46). Future research should further examine the proposed quantitative coding scheme (e.g., using Exploratory Factor Analysis); it is possible that some components (e.g., empathy for pain) of belief categories may better fit into different categories. Fourth, the participation in a research study at a pain research laboratory may have influenced what parents chose to share during the interviews. Finally, given the cross-sectional nature of the study, the directionality of findings cannot be inferred. Parent reminiscing behaviors may form parents' beliefs regarding reminiscing functions. Future research needs to include longitudinal investigations to examine the directionality of the association between parental beliefs and reminiscing behaviors.

## Conclusion

The current study identified key beliefs that parents hold with regards to reminiscing with their children about past pain. Three key beliefs (i.e., reminiscing to process past pain, reminiscing as a learning tool, and avoiding reminiscing) were related to parent reminiscing behaviors. The more parents endorsed avoiding reminiscing with their children, the less they used elaborative reminiscing elements. In contrast, the more parents endorsed reminiscing to process past pain, the more they talked about pain-associated emotions. Parents also were more likely to endorse reminiscing to process past pain if they had older children. Given the role of parent-child reminiscing about past pain in children's memories for pain and empathy behaviors toward others' pain, further investigations of parental beliefs about the functions of pain reminiscing are warranted.

## Data Availability Statement

The raw data supporting the conclusions of this article will be made available by the authors, without undue reservation.

## Ethics Statement

The studies involving human participants were reviewed and approved by Conjoint Health Research Ethics Board (CHREB) of the University of Calgary. Written informed consent to participate in this study was provided by the participants' legal guardian/next of kin.

## Author Contributions

All authors listed have made a substantial, direct, and intellectual contribution to the work and approved it for publication.

## Funding

This work was supported by the Social Sciences and Humanities Research Council Insight Development Grant (Grant No. 430-2017-00089) awarded to MN. MN holds the Killam Memorial Emerging Leader Chair. MP was supported by the Alberta Strategy for Patient-Oriented Research Graduate Studentship, the University of Calgary Eyes High Doctoral Scholarship, Alberta Innovates Graduate Studentship in Health Innovation, Frederick Banting and Charles Best Canada Graduate Scholarships, and Izaak Walton Killam Doctoral Scholarship. TL was supported by Canada Graduate Scholarship Masters Award.

## Conflict of Interest

The authors declare that the research was conducted in the absence of any commercial or financial relationships that could be construed as a potential conflict of interest.

## Publisher's Note

All claims expressed in this article are solely those of the authors and do not necessarily represent those of their affiliated organizations, or those of the publisher, the editors and the reviewers. Any product that may be evaluated in this article, or claim that may be made by its manufacturer, is not guaranteed or endorsed by the publisher.
