# Supplementary material for: Let's (Not) Talk About Pain: Mothers' and Fathers' Beliefs Regarding Reminiscing About Past Pain
Source: Front Pain Res (Lausanne). 2022 Apr 28;3:890897. doi: 10.3389/fpain.2022.890897 (PMC9095907; doi:10.3389/fpain.2022.890897)
Supplement: Supplementary file 1 [file Table_1.DOCX]

Supplementary Material

# 1 Beliefs Regarding the Functions of Reminiscing Qualitative Interview

In addition to asking you and your child talk about past, we also want to know your opinion on reminiscing – and specifically reminiscing and talking about past distressing events, like physical pain and emotional pain/sadness.

1. The research that we have done so far shows that parents and children tend to talk about past pain versus sadness quite differently. Some parents are more willing and more comfortable talking about past events that involved sadness – but not pain. What are your thoughts about that?
2. How do you and your child talk about past painful events or pain in general in everyday life? Why/why not do you talk about past pain?
3. In what situation/for what reason would you talk about past painful experiences?
4. What are the pros and cons of talking about past events that involved pain?
5. Does your child ever start conversations about past painful experiences? In what situations? Why do you think your child starts the conversation?

**2 Beliefs Regarding the Functions of Reminiscing Quantitative Coding Scheme**

**2.1 Non-functional**

- *Null.* Statements where there is no distinct idea that has not already been expressed. Statements where parents only mention when they talk about the past without a clear reason *why* (e.g., “In the car”).

**2.2 Reminiscing to Process Past Pain**

- *Contextualizing.* To put a painful event into perspective for their child. Using a past painful event as a way to help their child understand a current or future painful experience. You were okay last time and you will be okay now. Get over smth.
- *Normalizing.* To frame a painful event as normal, explaining that pain happens to everyone and it is manageable. Not a taboo.
- *Coping skills.* Using a past painful event to remind children of how they coped in the past. Discussing ways to better manage a future painful event. Reminiscing to demonstrate the child’s resilience and self-efficacy.
- *Fostering positive/healthy emotions.* To help their child process pain-related emotions. To prevent their child from suppressing negative emotions. Teaching them emotion-regulation skills. Help them feel less scared. Okay to talk about it. Understand why it happened.
- *Empathy development.* To teach their children empathy for others’ pain.

**2.3 Reminiscing as a Learning Tool**

- *Teaching to prevent pain.* To use a past painful event as a learning tool or to reinforce a lesson that would prevent the child from hurting themselves again. Focuses on *actions* that led to pain. For example, “Remember when you tripped here because you weren’t paying attention while you were running?” Learning a lesson. Trigger to remember pain.
- *Warning/threatening.* Invoking memories of *pain sensations* to discourage unsafe/unwanted behaviours. For example, “Remember when you fell off the slide and hurt your arm?”
- *Preparing for future pain.* To help their child feel ready for a future predictable event (e.g., vaccines), or common unpredictable events (e.g., minor injuries). Temporary pain.

**2.4 Avoiding Reminiscing about Past Pain**

- *Fear and traumatization.* Discussing a past painful event will trigger fear and anxiety in their child, cause their child to dwell on the pain or entrench the trauma of the initial experience. Reminiscing may discourage child from engaging in normal play/activities because of pain-related fear. Feeling pain again.
- *The lack of value of reminiscing.* No reason to revisit or process past painful events. For example, “It’s not something I really do or really necessarily see much point in?”
- *Focusing on the present.* May discuss pain when it happens/as needed, but feel the need to ‘move on’, ‘keep things positive’, or ‘live in the moment’.
- *Protectiveness.* Avoiding reminiscing to shelter their child, reduce their child’s exposure to pain, etc. For example, “I don’t really want him to feel those things.” Pain is there and I can’t fix it.
